# Newly Developed Graft Failure Detected Using Computed Tomography Within 1 Year After Coronary Artery Bypass Grafting Surgery: One Single-Center Experience

**DOI:** 10.3389/fcvm.2022.779015

**Published:** 2022-01-31

**Authors:** Zhaoshui Li, Youjin Qiao, Wei Sheng, Yifan Chi

**Affiliations:** ^1^Cardiac Surgery Department, Qingdao Medical College of Qingdao University, Qingdao, China; ^2^Cardiac Surgery Department, Qingdao Hiser Hospital Affiliated to Qingdao University, Qingdao, China; ^3^Cardiac Surgery Department, Qingdao Municipal Hospital Affiliated to Qingdao University, Qingdao, China

**Keywords:** computed tomography angiography, transit-time flow measurement, coronary artery bypass grafting, newly developed graft failure, adverse cardiovascular events

## Abstract

**Background:**

Newly developed graft failure negatively affects the short- and long-term outcomes of patients who experience coronary artery bypass grafting (CABG) surgery. This study explored the value of transit time flow measurement (TTFM) parameters for predicting the risk of newly developed graft failure that occurs within 1 year after CABG, as well as investigated the relationship between newly developed graft failure and adverse cardiovascular events.

**Methods:**

A total of 134 patients who underwent CABG and had CT angiography (CTA) data (1 year post-operatively) were divided into two groups: the patient group, in which patients did not have newly developed graft failure, and the occluded group, in which patients developed newly developed graft failure between 1 and 12 months after CABG. The patency rate of grafts in different targets was analyzed. The correlations between graft failure and TTFM parameters and between graft failure and the occurrence of adverse cardiovascular events were investigated.

**Results:**

The overall rate of newly developed graft failure was 7.2%, the venous graft failure was 10.8%, and the arterial graft failure was 0.7%. The occluded group had a higher pulse index (PI) (2.9 vs. 2.4, *P* = 0.007), a lower mean graft flow (MGF) (20 vs. 25 ml/min, *P* = 0.028), and a lower diastolic flow fraction (DF) (63.5 vs. 70%, *P* = 0.019) than the patent group. The cut-off value for predicting newly developed graft failure was PI > 2.75 (*P* = 0.007), MGF < 23.5 ml/min (*P* = 0.03), and DF < 65.5% (*P* = 0.019). Compared with the patent group, the newly developed graft failure group had higher rates of recurrent angina (13.6 vs. 0.9%, *P* = 0.0014) and revascularization intervention (9.1 vs. 0% *P* = 0.026). However, there were no differences in death, cardiac death, myocardial infarction, and cerebral infarction after CABG operation between these two groups (*P* > 0.05).

**Conclusions:**

A high PI and low MGF and DF are risk factors for newly developed graft failure. The patients with newly developed graft failure had higher rates of recurrent angina and revascularization intervention. TTFM parameters may be used to predict the occurrence of newly developed graft failure in patients after CABG surgery.

## Background

The first coronary artery bypass graft surgery in patients with coronary heart disease in 1961 was carried out by Robert Goetz (1). Since then, coronary artery bypass graft surgery (CABG) has remained a significant revascularization treatment strategy for coronary heart disease (CHD) (2). However, one adverse outcome of CABG is graft failure. Previous studies have shown that graft failure, defined as newly developed graft failure as evaluated by coronary angiography (3–6), develops rapidly in the first year after CABG and negatively affects short- and long-term outcomes of patients. Therefore, functional evaluation of anastomoses is crucial (7, 8). Currently, several methods are used in the clinic to assess graft function. Several studies have suggested that the low-invasive method of cardiac CT angiography (CTA) is a better alternative to coronary artery angiography for assessing early grafts (9–11).

Transit-time flow measurement (TTFM) is a fast and reliable technique for the intraoperative evaluation of grafts and is thus frequently applied during CABG (12). The value of TTFM in predicting the function of early postoperative grafts has been previously examined, but the findings are not conclusive. For example, a more recent Randomized On-Off Bypass (ROOBY) trial showed no association between TTFM and graft patency and clinical outcomes (13), but another study suggested that TTFM should be used as a routine procedure in patients following CABG (14). Moreover, many studies primarily checked grafts between 3 and 12 months following CABG (15, 16), and a few studies examined the correlation between TTFM parameters and the newly developed graft failure that occurred between 1 and 12 months after surgery.

This study investigated the predictive value of TTFM parameters for newly developed graft failure occurring within 1 year after successful CABG, as determined by CTA, and investigated the relationship between newly developed graft failure and adverse cardiovascular events (ACEs) during postoperative follow-up.

## Materials and Methods

### Study Population

Data of patients who underwent isolated CABG were retrospectively collected between July 1, 2018, and December 31, 2019, from the database at Qingdao Municipal Hospital Affiliated with Qingdao University. A total of 176 patients underwent CABGs. Among these patients, 14 with no CTA data and 28 who developed graft failure before discharge were excluded from this study. Hence, this study enrolled a total of 134 patients, who were divided into two groups: the patent group, which was defined as having patent anastomoses, and the occluded group, which included newly developed graft failure defined by at least one occluded anastomosis. The Institutional Review Board and Ethics Committee at our hospital approved the study protocol. Informed consent from patients was waived owing to the retrospective nature of this study. The use of data at the time of operative consent was approved.

### Surgical Methods

Coronary artery bypass graft surgery (CABG) was performed on all patients through a median full sternotomy. A tissue stabilizer (Octopus, Medtronic Corporation, Minneapolis, MN, USA) was used to stabilize the target coronary arteries. An intra-coronary shunt (Medtronic Corporation, Minneapolis, MN, USA) was used during off-pump CABG. In on-pump CABG, cardiopulmonary bypass was generated following standard cannulation of the ascending aorta and cavo-atrial cannulation. The surgeon measured each bridge first, and when the patient's blood pressure was relatively stable at (90–120)/(70–90) mmHg and did not require circulatory support or medication, the blood pressure was measured three times, and the average was used for this study. All distal anastomoses were carried out after cardiac arrest.

### Transit-Time Flow Measurement (TTFM)

The VeriQ system TTFM device (MediStim Inc., Oslo, Norway) was used to measure TTFM parameters, which included the diastolic flow fraction (DF), the pulse index (PI), and the mean graft flow volume (MGF). The criteria for assessment of satisfactory blood flow parameters were as follows: the morphology of the blood flow waveform was reproducible and stable; MGF > 15 ml/min and PI < 5. If a sufficient graft flow was not obtained, grafts were revisited to rule out technical complications. In latter cases, the TTFM parameters that were collected at the last measurement were used.

### CT Angiography (CTA)

Patients who underwent CABG without contraindications underwent cardiac CTA before discharge. The location, number, and patency of bypass grafts were assessed using Axial, 3D multi-planar, and volume-rendered reconstructed images. Two radiologists assessed graft patency. An occluded graft was defined as one that had no visualization or had visualization as a stump-like structure. CTA was rechecked 4–25 days after surgery and before discharge. The median CTA after surgery was 15 days, and the median of CTA postoperative recheck was 382 days.

### Adverse Cardiovascular Events (ACEs)

Adverse cardiovascular events (ACEs) included recurrent angina, revascularization intervention, cardiac death, myocardial infarction, and cerebral infarction.

### Statistical Analysis

Abnormally distributed continuous variables are presented as the median and interquartile range (IQR). Nominal and categorical variables are expressed as proportions (%) and absolute numbers. Numerical data were compared using the Mann–Whitney *U*-test between two groups. Pearson's χ^2^ test or Fischer's exact test was used to comparing binary data as appropriate. Uni-and multi-variate models using Logistic regression and odds ratios (ORs) were used to analyze the TTFM parameters to define the independent predictors of risk of graft failure. The optimal cutoff values of PI, MGF, and DF were determined using the receiver operating characteristic (ROC) curve to predict newly developed graft failure. A *P* < 0.05 was considered statistically significant. All analyses were performed with SPSS version 23 (SPSS., Chicago, IL, USA) and GraphPad Prism 5 (GraphPad Software Inc., San Diego, CA, USA).

## Results

A total of 134 patients were included in this study. Among these 134 patients, 22 (16.41%) had newly developed graft occlusion within 1 year after CABG. Demographic and baseline clinical characteristics of patients in both the patent and the occluded groups are shown in Table 1. There were no differences between these two groups with regard to age, gender, and the vast majority of baseline clinical characteristics. However, the occluded group had twice the amount of left main disease (45.5 vs. 21.4%, *P* = 0.03).

**Table 1:** Baseline patient characteristics of the patent group and the occluded group.

	**Patent (112)**	**Occluded (22)**	***P***
Age	63.0 (57.0–69.0)	59.0 (56.0–68.3)	0.567
Female (*n*, %)	26 (23.2)	6 (27.3)	0.785
BMI	25.4 (23.0–27.7)	26.1 (24.3–27.8)	0.505
Hypertension (*n*, %)	79(70.5)	12 (54.5)	0.210
Diabetes (*n*, %)	46(41.1)	5 (22.7)	0.149
Insulin treatment	14 (12.5)	2 (9.1)	1.000
Hyperlipidemia (*n*, %)	56 (50.0)	10 (45.5)	0.817
Previous stroke (*n*, %)	9 (8.1)	4 (18.2)	0.229
Smoking (*n*, %)	32 (28.6)	6 (27.3)	1.000
PVD (*n*, %)	15 (13.4)	4 (18.2)	0.517
Ventricular aneurysm (*n*, %)	4 (3.6)	0 (0.0)	1.000
Previous MI (*n*, %)	22 (19.6)	1 (4.5)	0.122
PCI	18(16.2)	4 (18.2)	0.761
Arrhythmia (*n*, %)	3 (2.7)	1 (4.5)	0.516
AMI (*n*, %)	28 (25.0)	7 (31.8)	0.596
Diseased vessels	3.0 (2.0–3.0)	3.0 (3.0–4.0)	0.912
Left main disease (*n*, %)	24 (21.4)	10 (45.5)	0.030
NYHA (*n*, %)			0.361
I	0 (0.0)	1 (4.5)	
II	87 (77.7)	17 (77.3)	
III	24 (21.4)	4 (18.2)	
IV	1 (0.9)	0 (0.0)	
LVEF (%)	64.1 (56.6–68.7)	64.6 (59.2–72.3)	0.239
LVEF <40% (*n*, %)	2 (1.8)	1 (4.5)	0.421
EuroSCORE II	1.0 (0.8–1.5)	0.9 (0.6–1.6)	0.886
Triglycerides	1.5 (1.1–2.0)	1.4 (1.2–1.8)	0.996
PLT	195.0 (172.0–238.3)	218.0 (175.5–273.8)	0.091
Preoperative creatinine (μmol/ml, ±s)	71.0 (61.3–84.8)	66.5 (57.8–82.0)	0.818

*BMI, body mass index; COPD, chronic obstructive pulmonary disease; PVD, peripheral vascular diseases; PCI, percutaneous coronary intervention; AMI, acute myocardial infarction; NYHA, New York Heart Association; LVEF, left ventricular ejection fraction; LVEDd, left ventricular end-diastolic dimension; PLT, platelet count*.

In addition, there were no differences in baseline procedural characteristics and postoperative complications between the patent and occluded groups (*P* > 0.05; Table 2).

**Table 2:** Baseline procedural characteristics of the patent group and the occluded group.

**Items**	**Patent (112)**	**Occluded (22)**	***P***
On-pump	59 (52.7)	14 (63.6)	0.483
Vein harvest technique			0.283
Bridge	3 (2.7)	2 (9.1)	
Endoscopic	20 (18.0)	2 (9.1)	
Open	77 (69.4)	18 (81.8)	
No-touch	3 (2.7)	0 (0.0)	
Proximal anastomosis technique			0.805
PAC	26 (23.4)	5 (22.7)	
Anastomosis device	54 (48.6)	13 (59.1)	
SAC	26 (23.4)	4 (18.2)	
No-touch	5 (4.5)	0 (0.0)	
Sequential graft	53 (47.7)	8 (36.4)	0.359
Composite graft	7 (6.4)	2 (9.1)	0.645
IABP			0.178
No-use	101 (91.0)	19 (86.4)	
Pre-operation	5 (4.5)	1 (4.5)	
Intra-operation	3 (2.7)	2 (9.1)	
Post-operation	2 (1.8)	2 (0.0)	
Number of vessel conduits	3.0 (3.0–4.0)	3.0 (2.0–4.0)	0.392
Bleeding(ml)	500.0 (350.0–800.0)	500.0 (300.0–725.0)	0.305
Mechanical ventilation time(h)	12.0 (8.0–18.0)	14.5 (7.8–18.0)	0.190
Operation time(min)	270.0 (230.0–300.0)	270.0 (227.5–302.5)	0.090
Antiplatelet strategy			0.279
Aspirin	61 (54.4)	14 (63.6)	
Clopidogrel	4 (3.6)	1 (4.5)	
Dual- antiplatelet	47 (42.0)	7 (31.8)	
New POAF (*n*, %)	32 (28.8)	5 (22.7)	0.615
CRRT (*n*, %)	3 (2.7)	0 (0.0)	1.000

*IABP, intra-aortic balloon pump; LIMA, left internal mammary artery; SVG, saphenous vein grafting; PI, pulse index; MGF, mean graft flow; DF, diastolic flow fraction; POAF, postoperative atrial fibrillation; CRRT, continuous renal replacement therapy; PAC, partial aorta clamp; SAC, single aorta clamp*.

Detailed results of CTA examination 1 year after CABG are shown in Table 3. The grafts were divided into two groups: the patent group and the occluded group. Among the 375 grafts, the patent group had 348 grafts (92.8%), and the occluded group had 27 grafts. The overall rate of newly developed graft failure was 7.2%, the venous graft failure was 10.8%, and the arterial graft failure was about 0.7%. The composite graft failure was 15.4%. The results showed the rate of newly developed venous graft failure was significantly higher than that of newly developed arterial graft failure (10.8 vs. 0.7%, *P* = 0), and that the failure rate of the composite graft was significantly higher than that of arterial graft (15.4 vs. 0.7%, *P* = 0.018). Additionally, the patency of the venous graft in the circumflex and right coronary system was worse than that in the anterior descending system.

**Table 3:** Details of CTA examination at approximately 1 year after CABG.

	**Strategy**	**Graft failure (*N*)**	**Graft failure rate (%)**	***P***
*In-situ* LIMA (128)	LIMA-LAD/D/RAMUS (125)	1		
	LIMA-LCX/OM (3)	0	0.0	
				1.000
*In-situ* RIMA (9)	RIMA-D/LAD (6)	0	0.0	
	RIMA-RCA (3)	0	0.0	
				1.000
AO-SVG (161)	AO-SVG-LAD/D/RAMUS (40)	1	0.25	
	AO-SVG-LCX/OM (42)	8	19.0	
	AO-SVG-PL/PDA/RCA (79)	11	13.9	
				0.053
AO-SVG sequential (62)		4		
AO-RA (6)	AO-RA-LAD/D (1)	0	0	
	AO-RA-LCX/OM (1)	0	0	
	AO-RA-PDA/PL/RCA (4)	0	0	
				1.000
Composite grafting (9)	AO-SVG-Y-RIMA/LIMA (5)	1		
	LIMA/RIMA-Y-SVG (4)	1		
Total		27/375	7.2	
Venous		24/223	10.8	
Arterial		1/143	0.7	
Composite grafting		2/13	15.4	
				0.000

*LAD, left anterior descending artery; DIAG, diagonal branch; PDA, posterior descending artery; PL, posterolateral artery; RCA, right coronary artery; OM, obtuse marginal; LCX, left circumflex artery*.

**Figure 1:**
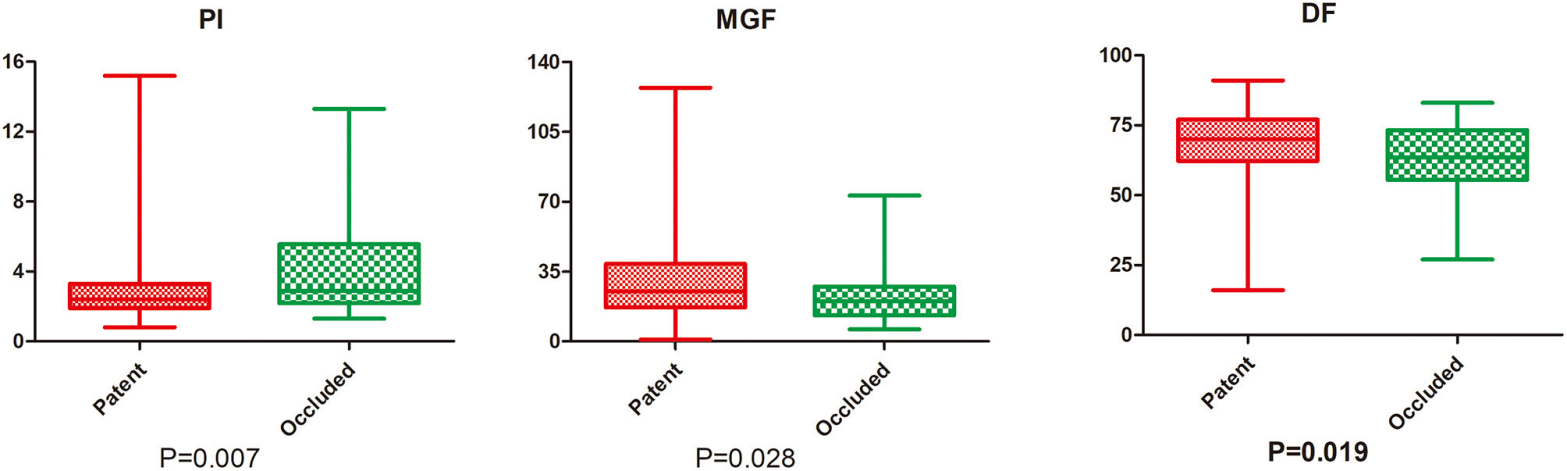
ROC analysis of newly developed graft failure. PI, pulse index; MGF, mean graft flow; DF, diastolic flow fraction.

**Figure 2:**
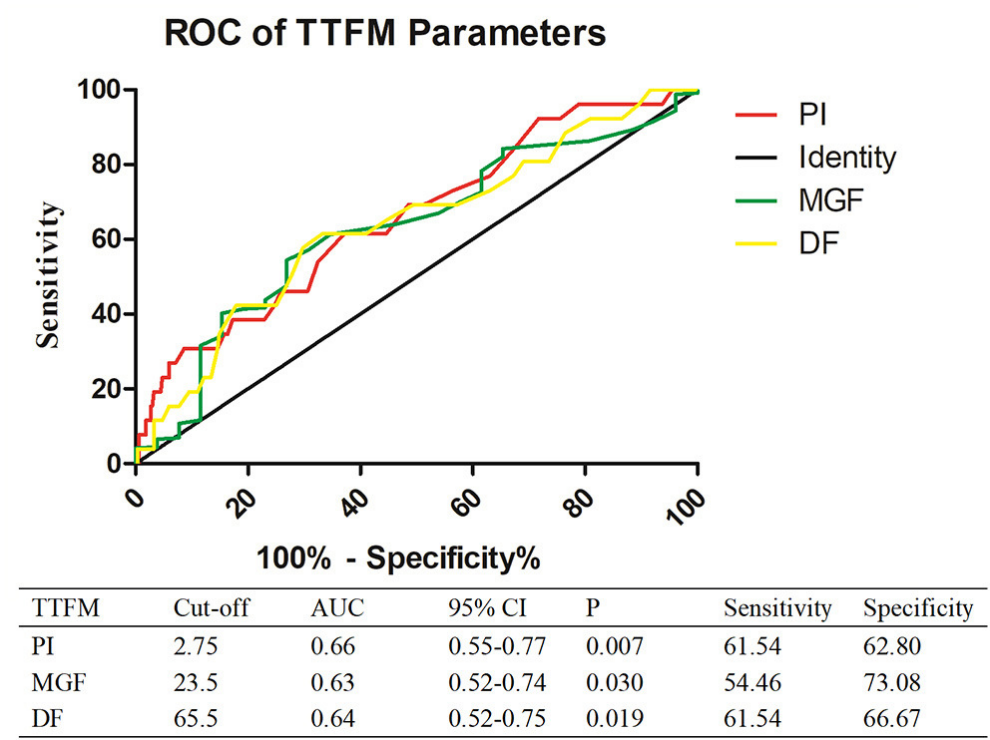
Comparison of TTFM parameters between the patent and occluded groups.

The comparison of parameters between the occluded and patent groups is shown in Figure 1. The occluded group had a higher PI (2.9 vs. 2.4, *P* = 0.007), a lower MGF (20 vs. 25 ml/min, *P* = 0.028), and a lower DF (63.5 vs. 70%, *P* = 0.019) than the patent group. ROC curve analysis showed that the cut-off value for predicting the overall newly developed graft failure was a PI > 2.75 (*P* = 0.007), a MGF < 23.5 ml/min (*P* = 0.03), and a DF < 65.5% (*P* = 0.019; Figure 2).

In addition, compared with the patent group, the occluded group had higher rates of recurrent angina (13.6 vs. 0.9%, *P* = 0.0014) and re-vascularization intervention (9.1 vs. 0% *P* = 0.026). However, there were no differences with regard to death, cardiac death, myocardial infarction, and cerebral infarction after operation between these two groups (*P* > 0.05; Table 4).

**Table 4:** Details of CTA examination at approximately one year after CABG.

**Item**	**Patent (112)**	**Occluded (22)**	***P***
Death	1 (0.9)	0 (0.0)	1.000
Cardiac death	0 (0.0)	0 (0.0)	NA
AMI	1 (0.9)	0 (0)	1.000
Postoperative angina	1 (0.9)	3 (13.6)	0.014
Reintervention	0 (0.0)	2 (9.1)	0.026
Cerebral infarction	2 (1.8)	0 (0.0)	1.000

*AMI, acute myocardial infarction*.

## Discussion

Coronary artery bypass graft surgery (CABG) surgery is the most frequently performed procedure worldwide (17). However, some patients have angina pectoris after surgery, which may be related to inadequate revascularization treatment or newly developed graft failure. In addition to pharmacological intervention and general follow-up examinations for patients after CABG, few patients have received CTA to estimate graft patency. Previous studies have reported that grafts fail most rapidly in the first year after CABG (3–6), defined as newly developed graft failure. Newly developed graft failure may negatively affect the short- and long-term outcomes of the patients, and patients with newly developed graft failure may develop symptoms when exercising due to insufficient blood flow to the myocardial tissue. As a low-invasive technique, cardiac CTA has made graft quality evaluation easier and reliable (7, 8).

In our center, CTA examination is routinely performed postoperatively on patients to determine if any newly developed graft failure 1–12 months after CABG. In the present study, we investigated the predictive value of TTFM parameters for newly developed graft failure occurring within 1 year after successful CABG, as determined by CTA, and investigated the relationship between newly developed graft failure and ACEs during postoperative follow-up. We excluded patients with grafts that failed before discharge. We found that the overall newly developed graft failure was 7.2% and that the venous graft failure was 10.8%, which were consistent with the results reported in previous studies (18, 19). However, the arterial graft failure was only 0.7%, which was lower than that previously reported (20). This difference could be attributed to the fact the placement of arterial grafts. In our study, the grafts were mainly in the left internal mammary artery (LIMA). In the subgroup analysis, the newly developed graft failure rates of composite grafts were significantly higher than the arterial grafts. Although arterial composite grafts have been demonstrated to be effective and safe for revascularization, studies evaluating the efficacy and safety of composite grafts using saphenous veins have produced conflicting results (19, 21). One study suggested that using a saphenous vein composite graft could steal flow from the stem graft and result in suboptimal short-term stem patency. Thus, the IMA graft was not recommended (22). In the present study, composite grafts including arterial conduits and saphenous vein conduits were confirmed to be a risk factor of the short-term patency. However, our study had a small sample size, and future studies with large cohorts are needed to further corroborate our findings.

Some clinical studies have suggested that TTFM is a useful technique to evaluate graft function following CABG (23). However, some reviews in the field present conflicting conclusions due to heterogeneous data, technical limitations, and various optimal cutoff values (24, 25). In the present study, we investigated the value of TTFM parameters for predicting newly developed graft failure 1 and 12 months after CABG and determined the cut-off values of TTFM parameters. Consistent with the above observations, we found that PI, MGF, and DF were independent risk factors that affected the occurrence of newly developed graft failure, with the cut-off values being 2.75, 23.5, and 64.5%, respectively. PI was suggested as an appreciable predictor of graft quality in previous studies, with a suggested cutoff value of 3 or 5 (21, 22). We identified the cutoff value of MGF as 23.5, which was consistent with the >20 ml/min recommended by the manufacturer of the Instruments VeriQ TTFM device. In contrast with previous studies (26), we found that DF was higher in the patent group than in the occluded group. This finding may be related to the fact that the patent group had a higher proportion of arterial grafts in the present study. Arterial grafts have high arterial wall elasticity, and there is higher blood flow through the arterial grafts during diastole. Additionally, in the present study, a high percentage of patent grafts was frequently implanted in the left anterior descending (LAD) artery, which has the largest vascular bed. Therefore, these grafts may also increase DF (27, 28). In addition, the occluded group had a higher rate of recurrent angina and re-vascularization intervention than the patent group, consistent with previous findings (29, 30). These findings indicate that newly developed graft failure should be given more attention and effective remedial measures should be in place that can reduce the incidence of ACEs in patients.

## Conclusions

We reported in this study that the overall rate of newly developed graft failure between 1 and 12 months after CABG was 7.2%, the venous graft failure was 10.8%, and the arterial graft failure was 0.7%. A high PI and low MGF and DF are risk factors for newly developed graft failure. Patients with newly developed graft failure have higher rates of recurrent angina and re-vascularization intervention. Our findings suggested that TTFM parameters may be used to predict the occurrence of newly developed graft failure in patients after CABG surgery.

## Data Availability Statement

The raw data supporting the conclusions of this article will be made available by the authors, without undue reservation.

## Author Contributions

ZL conceived the study and participated in its design and coordination. YQ, WS, and YC helped to draft the manuscript. All authors have read and approved the final manuscript.

## Conflict of Interest

The authors declare that the research was conducted in the absence of any commercial or financial relationships that could be construed as a potential conflict of interest.

## Publisher's Note

All claims expressed in this article are solely those of the authors and do not necessarily represent those of their affiliated organizations, or those of the publisher, the editors and the reviewers. Any product that may be evaluated in this article, or claim that may be made by its manufacturer, is not guaranteed or endorsed by the publisher.

## Abbreviations

LIMA: left internal mammary artery
LAD: left anterior descending artery
CABG: coronary artery bypass graft surgery
TTFM: transit-time flow measurement
PI: pulse index
MGF: mean graft flow
DF: diastolic flow fraction
SVG: great saphenous vein
CTA: computed tomography angiography.
